# Metabolic and senescence characteristics associated with the immune microenvironment in ovarian cancer

**DOI:** 10.3389/fendo.2023.1265525

**Published:** 2023-11-22

**Authors:** Jian Xiong, Yiyuan Fu, Jiezheng Huang, Yibin Wang, Xiaolong Jin, Xiaoyan Wan, Liu Huang, Zheng Huang

**Affiliations:** Department of Obstetrics and Gynaecology, Guangzhou Women and Children’s Medical Center, Guangzhou Medical University, Guangzhou, China

**Keywords:** ovarian cancer, metabolism, senescence, immune microenvironment, therapeutic strategies

## Abstract

Ovarian cancer is a highly malignant gynecological cancer influenced by the immune microenvironment, metabolic reprogramming, and cellular senescence. This review provides a comprehensive overview of these characteristics. Metabolic reprogramming affects immune cell function and tumor growth signals. Cellular senescence in immune and tumor cells impacts anti-tumor responses and therapy resistance. Targeting immune cell metabolism and inducing tumor cell senescence offer potential therapeutic strategies. However, challenges remain in identifying specific targets and biomarkers. Understanding the interplay of these characteristics can lead to innovative therapeutic approaches. Further research is needed to elucidate mechanisms, validate strategies, and improve patient outcomes in ovarian cancer.

## 1 Introduction

### 1.1 Background and importance of ovarian cancer

Ovarian cancer represents a substantial health concern and stands as one of the predominant contributors to cancer-related mortality among women on a global scale. The disease is characterized by its aggressive nature, late-stage diagnosis, and limited therapeutic avenues. The ovaries, responsible for the production of eggs and female hormones, serve as the primary site for the development of ovarian cancer. Existing academic inquiries have provided valuable insights into several pivotal facets of ovarian cancer. Epidemiological investigations have identified diverse risk factors encompassing age, familial history of ovarian or breast cancer, specific genetic mutations such as BRCA1 and BRCA2, as well as hormone-related factors including early menarche and late menopause. These findings have facilitated comprehension of the etiological aspects and identification of high-risk populations, thereby enabling targeted screening and prevention strategies (1, 2).

Furthermore, extensive research has been dedicated to unraveling the molecular mechanisms underpinning the development and progression of ovarian cancer. Studies have divulged multiple genetic aberrations, notably TP53 mutations, MYC and HER2 gene amplification, and dysregulation of the PI3K/AKT and RAS/RAF/MEK/ERK signaling pathways. These molecular alterations contribute to heightened cell proliferation, inhibition of apoptosis, and augmented angiogenesis, ultimately culminating in tumor growth and metastasis (3, 4). The significance of early detection and precise diagnosis of ovarian cancer cannot be overstated. Unfortunately, the disease is frequently detected at advanced stages, characterized by extraneous spread beyond the confines of the ovaries. This occurrence primarily arises due to the absence of specific symptoms and effective screening methodologies. Academic research endeavors have been concentrated on developing and refining diagnostic techniques, such as transvaginal ultrasound, serum biomarkers like CA-125, and more recently, liquid biopsies and molecular profiling. These advancements aim to enhance early detection, bolster diagnostic accuracy, and facilitate personalized treatment strategies (5, 6).

Treatment options for ovarian cancer commonly encompass a combination of surgical intervention, chemotherapy, and targeted therapies. Academic research has played a pivotal role in evaluating the efficacy of diverse treatment modalities while also identifying novel therapeutic targets. The advent of poly (ADP-ribose) polymerase (PARP) inhibitors, for instance, has brought about a revolutionary transformation in ovarian cancer treatment, particularly among patients harboring BRCA mutations. Additionally, immunotherapy, including the utilization of immune checkpoint inhibitors, is currently under exploration as a promising avenue for bolstering the immune response against ovarian cancer cells (7, 8).

In conclusion, ovarian cancer represents a complex ailment bearing substantial clinical ramifications. Academic research has significantly contributed to our understanding of the disease’s etiology, molecular mechanisms, early detection, and treatment options. These advancements hold tremendous potential for improving patient outcomes, augmenting survival rates, and ultimately alleviating the burden associated with this devastating disease. The primary objective of our review is to elucidate the interplay of these characteristics and its potential to drive innovative therapeutic approaches. However, it’s crucial to acknowledge that further research is imperative. This research should focus on unraveling the underlying mechanisms, validating these strategies, and ultimately enhancing patient outcomes in the realm of ovarian cancer.

### 1.2 The role of immune microenvironment in ovarian cancer

The immune microenvironment is of paramount importance in the pathogenesis and advancement of ovarian cancer. Numerous academic studies have elucidated diverse facets of this intricate interplay between neoplastic cells and the immune system, thereby providing valuable insights into the mechanisms underlying immune evasion by tumors, infiltration patterns of immune cells, and immunotherapeutic modalities for ovarian cancer. Impairment of immunosurveillance, the process through which the immune system recognizes and eliminates cancer cells, is a common occurrence in ovarian cancer. Extensive scholarly investigations have unveiled multiple mechanisms contributing to immune evasion, encompassing the upregulation of immune checkpoint molecules, such as programmed cell death ligand 1 (PD-L1), on neoplastic cells, and the recruitment of immunosuppressive cell populations like regulatory T cells (Tregs) and myeloid-derived suppressor cells (MDSCs). These mechanisms foster an immunosuppressive microenvironment that facilitates tumor growth and metastasis (9).

Moreover, studies have underscored the significance of immune cell infiltration in ovarian cancer. Tumor-infiltrating lymphocytes (TILs), particularly cytotoxic CD8+ T cells, have demonstrated favorable associations with prognosis and enhanced survival rates in ovarian cancer patients. Conversely, the presence of immunosuppressive cells, such as Tregs and MDSCs, correlates with unfavorable clinical outcomes. The composition and functional status of immune cells within the tumor microenvironment serve as pivotal determinants of disease progression and therapeutic response (10). Furthermore, scholarly research has directed its focus towards harnessing the potential of the immune system through various immunotherapeutic strategies (11–13). Immune checkpoint inhibitors targeting the PD-1/PD-L1 axis and cytotoxic T lymphocyte-associated antigen 4 (CTLA-4) pathway have exhibited promising outcomes in clinical trials, with some patients displaying enduring responses. Combination therapies, entailing the simultaneous administration of immune checkpoint inhibitors, chemotherapy, or targeted agents, are being actively explored to augment treatment efficacy. In addition, the investigation of adoptive cell therapies, including chimeric antigen receptor (CAR) T cell therapy and tumor-infiltrating lymphocyte (TIL) therapy, is underway to evaluate their potential in the treatment of ovarian cancer (14).

A comprehensive comprehension of the dynamic interplay between the immune microenvironment and ovarian cancer has paved the way for personalized immunotherapeutic approaches. Biomarkers such as PD-L1 expression, TILs, and immune gene signatures have been extensively investigated to forecast treatment response and patient outcomes. Furthermore, scholarly research has shed light on the role of the gut microbiome in modulating systemic immune responses, highlighting its potential as a therapeutic target in ovarian cancer (15–18). To conclude, the immune microenvironment exerts a pivotal influence on ovarian cancer, encompassing tumor progression, immune evasion, and treatment response. Academic research has significantly deepened our understanding of the intricate interactions between neoplastic cells and the immune system. These findings have laid the foundation for the development of innovative immunotherapeutic strategies, holding great promise for enhancing outcomes in ovarian cancer patients.

## 2 Metabolic characteristics in the immune microenvironment

### 2.1 Regulation of immune cell metabolism in the tumor microenvironment

The immune microenvironment has unveiled the pivotal role of metabolic reprogramming in immune cell function. Metabolism plays a vital role in providing energy and biosynthetic precursors necessary for immune cell activation, proliferation, and effector functions. Comprehending the intricate regulation of immune cell metabolism is imperative for unraveling the complex dynamics of immune responses in diverse disease contexts, such as cancer. Among the key metabolic pathways influencing immune cell function, glycolysis stands out prominently. Scholarly investigations have demonstrated that upon activation, immune cells, including T cells, undergo a metabolic shift towards aerobic glycolysis, even under oxygenated conditions. Termed the Warburg effect, this metabolic transition enables immune cells to promptly generate ATP and biosynthetic intermediates to meet their energetic and biosynthetic demands. Crucial players in this process encompass glycolytic enzymes and transporters like hexokinase, glucose transporters (GLUTs), and lactate dehydrogenase (LDH) (19, 20).

Additionally, studies have pinpointed key signaling pathways governing immune cell metabolism. Notably, the mammalian target of rapamycin (mTOR) pathway integrates various signals, including nutrient availability, growth factors, and cytokines, to modulate immune cell metabolism and function. mTOR promotes glycolysis and anabolic processes while suppressing oxidative phosphorylation and catabolic processes in immune cells. Other significant regulators of immune cell metabolism, namely AMP-activated protein kinase (AMPK) and hypoxia-inducible factor 1 alpha (HIF-1α), respond to nutrient and oxygen availability, respectively (21, 22).

Furthermore, metabolic reprogramming in immune cells profoundly impacts immune cell differentiation and effector functions. For instance, effector T cells rely on glycolysis to sustain their robust proliferation and cytokine production, while Tregs preferentially employ oxidative phosphorylation and fatty acid oxidation to support their suppressive function. Metabolic checkpoints, such as the intracellular ATP to AMP ratio, as well as metabolic intermediates like acetyl-CoA and α-ketoglutarate, function as signaling molecules influencing immune cell fate and function (23, 24). Importantly, dysregulation of immune cell metabolism can contribute to immune dysfunction and disease progression. Within the tumor microenvironment, metabolic competition between tumor cells and infiltrating immune cells, along with the presence of immunosuppressive factors, can restrict immune cell metabolism and function. Tumor cells may deprive immune cells of nutrients, secrete metabolites with immunosuppressive effects, or upregulate immune checkpoint molecules that attenuate immune responses. Academic research has underscored the potential of targeting immune cell metabolism to restore immune cell function and augment anti-tumor immunity in cancer immunotherapy (25, 26).

In conclusion, academic research has shed light on the regulation of immune cell metabolism in the immune microenvironment. Understanding the metabolic requirements and adaptations of immune cells assumes paramount importance in deciphering the dynamics of immune responses and devising targeted immunotherapeutic strategies. Further investigations are warranted to elucidate the intricate mechanisms governing immune cell metabolism and its modulation in various disease contexts, including cancer.

### 2.2 Effects of tumor cell metabolism in the immune microenvironment, such as nutrient supply and growth signaling

**Figure 1:**
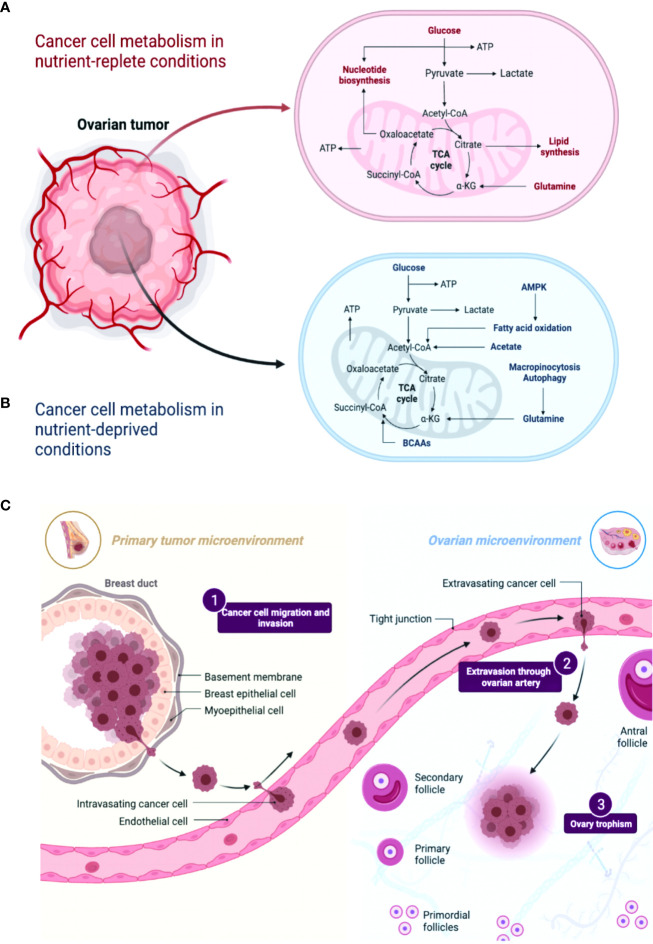
A comparison of metabolic pathways in ovarian cancer cells under conditions of nutrient abundance and nutrient deprivation revealed distinct metabolic alterations. **(A, B)** In nutrient-rich conditions, ovarian cancer cells tend to rely on glycolysis and exhibit increased glucose uptake and lactate production. Conversely, under nutrient-deficient conditions, these cells exhibit metabolic adaptations such as enhanced autophagy and utilization of alternative energy sources like fatty acids and amino acids. Understanding these metabolic differences may provide insights into novel therapeutic strategies targeting the specific metabolic vulnerabilities of ovarian cancer cells. **(C)** A comparison between the primary tumor microenvironment and the ovarian cancer microenvironment reveals distinct differences. In the primary tumor microenvironment, various cell types, including cancer cells, stromal cells, immune cells, and extracellular matrix components, interact to shape tumor progression. However, in the ovarian cancer microenvironment, additional factors like pericytes and specific inflammatory cytokines and chemokines contribute to its unique composition. These variations highlight the importance of considering the specific characteristics of the ovarian cancer microenvironment in understanding disease biology and developing targeted therapeutic approaches.

Tumor cell metabolism plays a pivotal role in shaping the immune microenvironment by influencing the availability of nutrients, signaling pathways associated with growth, and the functionality of immune cells (**Figure 1**). Extensive scholarly investigations have elucidated the intricate interplay between tumor cell metabolism and immune responses, shedding light on the mechanisms underlying immune evasion and proposing potential therapeutic approaches. Notably, one of the principal consequences of tumor cell metabolism is the alteration of nutrient availability within the immune microenvironment. Tumor cells exhibit heightened nutrient uptake, particularly glucose and amino acids, to support their rapid proliferation and survival. However, this heightened metabolic demand can deprive infiltrating immune cells of essential nutrients, thereby affecting their activation, proliferation, and effector functions. For instance, glucose competition can restrict the glycolytic capacity of immune cells, impairing their ability to mount an effective anti-tumor response (26, 27).

Furthermore, tumor cells can generate and release metabolites that shape the immune microenvironment. Enhanced glycolysis results in the accumulation of lactate, which suppresses immune cell function. Lactate hampers T cell activation, impairs cytotoxicity, and facilitates the expansion of immunosuppressive cell populations, including MDSCs and Tregs. In addition, tumor-derived metabolites such as kynurenine and adenosine exert immunosuppressive effects by inhibiting T cell proliferation and cytokine production (28, 29). Moreover, tumor cell metabolism influences the signaling pathways associated with growth within the immune microenvironment. Metabolic alterations in tumor cells can lead to the production of growth factors, cytokines, and chemokines that promote both tumor progression and modulation of immune cell behavior. For example, the activation of the mTOR pathway in tumor cells can induce the secretion of VEGF, which facilitates angiogenesis and tumor growth (30). The study investigated the role of mTOR, AP-1, and VEGF in vascular endothelial cell proliferation, showing mTOR downregulation and VEGF upregulation as potential strategies to inhibit restenosis and maintain normal vascular endothelial cell growth after PCI or CABG. This suggests a crucial role for the mTOR/AP-1/VEGF pathway in regulating vascular endothelial cell growth (30). Additionally, tumor cell metabolism can shape the composition and functionality of immune cell populations by releasing factors such as transforming growth factor-beta (TGF-β) and interleukin-10 (IL-10), which suppress immune responses (31, 32).

Moreover, metabolic crosstalk between tumor cells and immune cells can influence the polarization of immune cells and impact functional outcomes. Tumor-associated macrophages (TAMs), for example, undergo metabolic reprogramming in response to tumor-derived signals, leading to their polarization toward an immunosuppressive phenotype (33). This study provides a novel mechanism by which macrophages within a pre-metastatic niche acquire their immunosuppressive phenotype and identifies an important link among exosomes, metabolism, and metastasis (33). Tumor cells also have the capacity to modulate the metabolism of dendritic cells, impairing their ability to present antigens and promoting immune tolerance. These metabolic alterations collectively contribute to the establishment of an immunosuppressive microenvironment that facilitates tumor growth and evades immune surveillance (24, 34). Therefore, a comprehensive understanding of the effects of tumor cell metabolism in the immune microenvironment is imperative for the development of effective therapeutic strategies. Targeting tumor cell metabolism, including glycolysis or specific metabolic pathways, holds promise for overcoming immune suppression and augmenting anti-tumor immunity. Researchers are exploring the combination of metabolic interventions with immunotherapies, such as immune checkpoint blockade or adoptive cell therapies, as a potential approach to enhance treatment outcomes (25, 35).

Tumor cell metabolism exerts significant and multifaceted effects on the immune microenvironment, impacting nutrient availability, growth signaling, and immune cell function. Academic research has considerably deepened our understanding of these complex interactions and revealed potential avenues for therapeutic intervention. However, further studies are warranted to unravel the intricate mechanisms underlying the metabolic crosstalk between tumor cells and immune cells and to translate these findings into effective clinical strategies.

### 2.3 Effects of tumor cell metabolism on reprogramming of metabolic pathways in the immune microenvironment

Tumor cells demonstrate metabolic alterations that exert influences on nutrient availability, signaling pathways, and immune cell function, thereby contributing to the progression of tumors and the evasion of immune responses. Notably, a significant consequence of tumor cell metabolism is the reconfiguration of metabolic pathways in immune cells. Through various mechanisms, tumor cells possess the capability to modify the metabolic profiles of infiltrating immune cells, including T cells and macrophages. This reprogramming of immune cell metabolism results in a shift in their metabolic preferences, ultimately impacting their effector functions and fostering an immunosuppressive microenvironment. The Warburg effect, characterized by enhanced glycolytic metabolism, is frequently observed in tumor cells. Consequently, this metabolic phenotype leads to heightened glucose consumption and lactate production. The accumulation of lactate within the tumor microenvironment can induce extracellular acidification, consequently impairing immune cell function.

Additionally, tumor cells can induce metabolic reprogramming in immune cells through the release of immunosuppressive factors and metabolites. For instance, the production of adenosine by tumor cells inhibits T cell proliferation and function by impeding mitochondrial metabolism and attenuating immune responses. Tumor-derived kynurenine, a metabolite of tryptophan, can promote the differentiation of Tregs, thereby further contributing to immune suppression within the tumor microenvironment (36, 37). Moreover, tumor cells can modulate signaling pathways that are involved in immune cell metabolism. Activation of the mTOR pathway in tumor cells influences immune cell metabolism by altering nutrient availability and growth factor signaling. Factors derived from tumor cells, such as VEGF and TGF-β, can foster angiogenesis and immune cell polarization, thereby impacting metabolic reprogramming within the immune microenvironment (27, 32). Comprehensive comprehension of the effects of tumor cell metabolism on the reprogramming of metabolic pathways in the immune microenvironment is of paramount importance in the development of effective therapeutic strategies. Targeting the metabolic vulnerabilities of both tumor cells and immune cells holds immense potential for augmenting anti-tumor immune responses. Ongoing exploration of approaches like metabolic inhibitors, immunomodulatory agents, and combination therapies seeks to overcome immune suppression and enhance treatment outcomes.

The intricate interplay of metabolism between tumor cells and immune cells assumes a pivotal role in shaping immune responses and facilitating tumor progression. Further investigation into the multifaceted mechanisms underlying metabolic reprogramming will undoubtedly advance the development of innovative therapeutic interventions.

## 3 Senescence characteristics in the immune microenvironment

### 3.1 Characteristics of immune cell senescence

Immune cell senescence is characterized by irreversible cell cycle arrest and changes in functional properties, leading to immune dysfunction and compromised anti-tumor immune responses. Replicative senescence, a key characteristic of immune cell senescence, occurs due to cumulative cell divisions and telomere attrition. Senescent immune cells exhibit shortened telomeres, resulting in genomic instability and reduced proliferative capacity. Additionally, senescent immune cells display altered cell cycle regulators, including increased expression of cyclin-dependent kinase inhibitors (CDKIs) such as p16INK4a and p21Cip1/Waf1, contributing to cell cycle arrest (38, 39).

**Figure 2:**
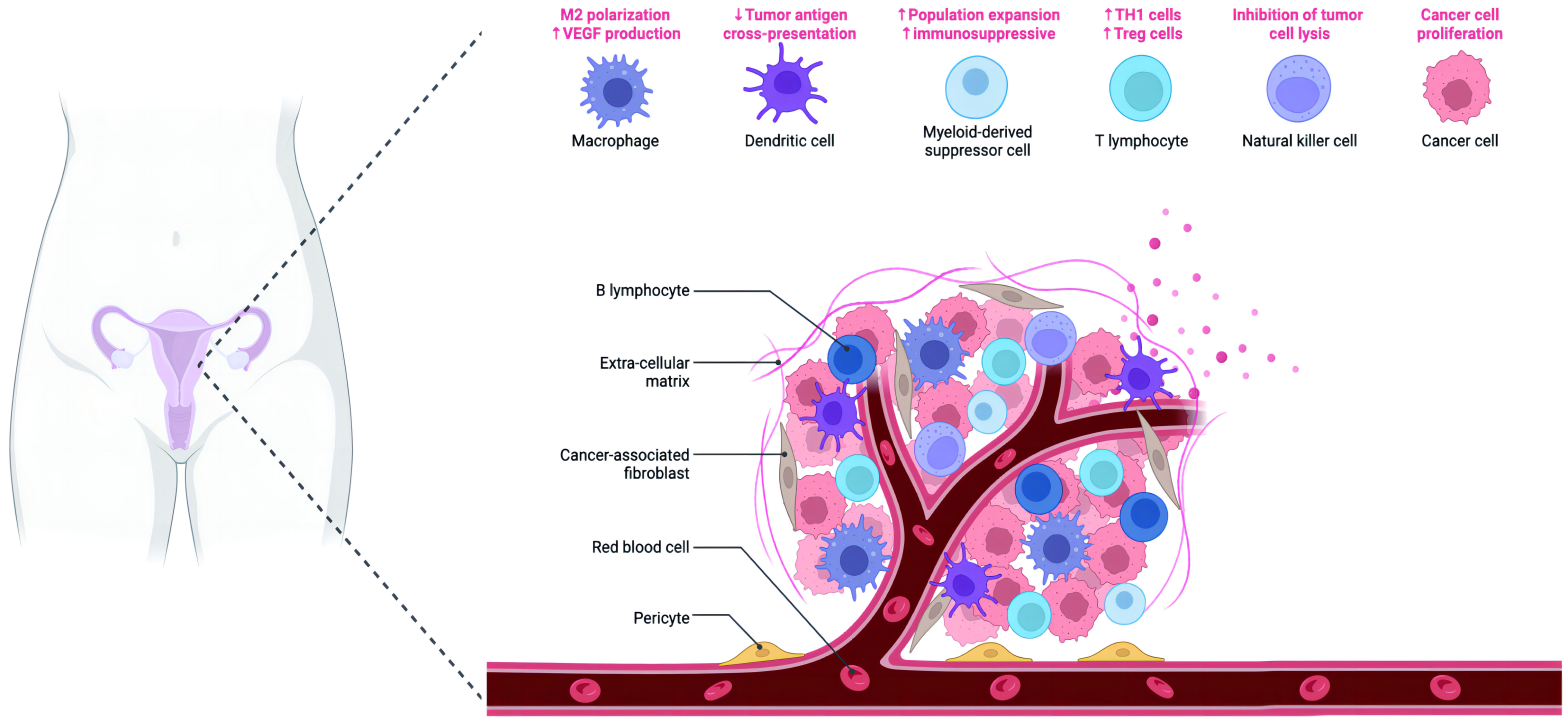
The figure depicts the intricate composition of the microenvironment in ovarian cancer. The ovarian cancer microenvironment encompasses various components, including ovarian cancer cells, stromal cells, immune cells, pericytes, and the extracellular matrix (ECM). The ECM comprises a complex network of inflammatory cytokines, chemokines, and other secreted molecules, contributing to the dynamic interactions within the microenvironment.

Functional alterations are observed in senescent immune cells, which significantly affect their ability to mount effective immune responses. For instance, senescent T cells exhibit decreased proliferation, impaired cytotoxicity, and compromised production of effector cytokines, such as interferon-gamma (IFN-γ) and tumor necrosis factor-alpha (TNF-α) (**Figure 2**). Senescent natural killer (NK) cells demonstrate reduced cytotoxicity and impaired cytokine secretion. Similarly, senescent dendritic cells (DCs) exhibit diminished antigen presentation capacity and altered cytokine production, thereby influencing the initiation and regulation of immune responses (40–42). The Senescence-Associated Secretory Phenotype (SASP) is a complex milieu of factors secreted by senescent cells, which play a pivotal role in the tumor microenvironment of ovarian cancer. SASP components include pro-inflammatory cytokines, chemokines, growth factors, and extracellular matrix remodeling enzymes. These factors influence both tumor and immune cells. Pro-inflammatory cytokines, such as IL-6 and IL-8, promote tumorigenesis, angiogenesis, and immunosuppression, fostering a favorable environment for ovarian cancer progression. Chemokines, like CXCL1, attract immune cells to the tumor site, impacting the antitumor immune response. Growth factors, such as EGF and TGF-β, contribute to cell proliferation and immune evasion. Understanding how SASP factors modulate the interactions between tumor and immune cells is critical for developing effective therapeutic strategies against ovarian cancer. The SASP contributes to chronic inflammation within the immune microenvironment, fostering tumor progression and immune dysfunction. Additionally, the SASP can induce senescence in neighboring cells, thereby perpetuating the senescence-associated phenotype (43, 44).

Furthermore, the tumor microenvironment and immunosuppressive factors influence immune cell senescence. Factors derived from tumors, such as TGF-β and IL-10, can expedite immune cell senescence and impair their function. Chronic exposure to inflammatory signals present in the tumor microenvironment also contributes to immune cell senescence through the activation of various pathways, including NF-κB and mTOR (45, 46). Gaining insights into the characteristics of immune cell senescence within the immune microenvironment is essential for the development of strategies to overcome immune dysfunction and enhance anti-tumor immune responses. Potential approaches to improving immunotherapeutic outcomes involve targeting senescent immune cells, modulating the SASP, and rejuvenating immune cell function. Furthermore, interventions addressing the root causes of immune cell senescence, such as senescence-inducing factors or DNA damage, hold promise for restoring immune cell functionality (47, 48).

Senescent immune cells exhibit altered proliferative capacity, functional impairments, and the acquisition of a pro-inflammatory secretory phenotype. Understanding the underlying mechanisms of immune cell senescence will pave the way for innovative therapeutic strategies aimed at restoring immune function and enhancing anti-tumor immune responses.

### 3.2 Effects of senescence on immune cell function

Senescent immune cells manifest functional alterations that actively contribute to immune dysfunction and compromised anti-tumor immune responses, thereby necessitating a comprehensive comprehension of these effects for the advancement of strategies aimed at invigorating immune cell function and enhancing immunotherapeutic approaches. A pivotal repercussion of senescence on immune cell function is the diminished proliferative capacity experienced by these cells. Senescent immune cells undergo an irreversible cell cycle arrest, thereby limiting their potential for expansion and impeding the efficacy of immune responses. This hampered proliferative capacity is primarily attributed to the upregulation of cell cycle inhibitors, notably p16INK4a and p21Cip1/Waf1, which hinder cell cycle progression (38). In addition to reduced proliferative capacity, senescent immune cells exhibit altered functional properties. For instance, senescent T cells display diminished cytotoxicity, impaired cytokine production, and a decreased ability to activate other immune cells. These functional deficiencies compromise the capacity of T cells to eradicate cancer cells and control tumor growth. Similarly, senescent NK cells demonstrate diminished cytotoxicity and compromised production of effector cytokines, thereby impairing their capacity to identify and eliminate target cells (40, 42).

Furthermore, senescence exerts an impact on the immunosurveillance capabilities of immune cells. Senescent immune cells demonstrate impaired antigen presentation capacity, consequently affecting T cell activation and the initiation of immune responses. DCs, which are crucial antigen-presenting cells, undergo functional alterations during senescence, leading to reduced antigen uptake, diminished expression of major histocompatibility complex (MHC) molecules, and altered cytokine production. Consequently, their ability to initiate and regulate immune responses is compromised (41, 49). Senescent immune cells also contribute to chronic inflammation within the immune microenvironment. Senescent cells secrete factors associated with the SASP, including pro-inflammatory cytokines, chemokines, and growth factors, which promote chronic inflammation, further hindering immune cell function and facilitating tumor progression. The SASP can induce senescence in neighboring cells, thereby propagating the senescent phenotype and perpetuating immune dysfunction (43).

Moreover, senescent immune cells can foster an immunosuppressive microenvironment. These cells express inhibitory receptors, such as PD-1, which interact with their ligands, such as PD-L1, resulting in immune cell exhaustion and impaired anti-tumor immune responses. Furthermore, the interaction between senescent immune cells and immunosuppressive cells, including Tregs and MDSCs, contributes to immune suppression within the tumor microenvironment (50). By understanding the effects of senescence on immune cell function, valuable insights into the mechanisms underlying immune dysfunction in aging and cancer can be obtained. Targeting senescent immune cells, modulating the SASP, and rejuvenating immune cell function emerge as potential therapeutic strategies for reinstating immune competence and enhancing anti-tumor immune responses.

### 3.3 Relationship between senescence and tumor progression

Senescence initially functions as a tumor-suppressive mechanism, known as oncogene-induced senescence (OIS), by halting the proliferation of premalignant cells triggered by oncogenic signals or DNA damage. Moreover, OIS promotes immune clearance of these cells, preventing the formation of fully malignant tumors. However, the SASP factors released by senescent cells can paradoxically promote tumor progression. SASP creates a pro-inflammatory microenvironment that supports tumor cell proliferation, survival, and angiogenesis through activation of signaling pathways such as NF-κB and STAT3 (51, 52).

Senescent immune cells, including senescent T cells and NK cells, exhibit compromised cytotoxicity and impaired cytokine production, impairing their ability to eliminate cancer cells. Furthermore, senescent immune cells secrete immunosuppressive factors that facilitate tumor immune evasion and promote tumor progression. Additionally, the accumulation of senescent cells within the tumor microenvironment contributes to tumor growth and therapy resistance. Senescent cells display metabolic reprogramming and enhanced survival mechanisms that promote their persistence in the tumor, while also acquiring stem cell-like properties through senescence-associated stemness. These properties contribute to tumor heterogeneity and therapy resistance (53, 54).

The phenomenon of senescence bypass further complicates the role of senescence in tumor progression. Tumor cells can evade senescence-induced growth arrest through various mechanisms, such as inactivation of tumor suppressor pathways or activation of telomerase. This enables continuous proliferation of tumor cells, thereby contributing to tumor growth and metastasis. Additionally, senescence influences the response to cancer therapy (54). Senescent cells within the tumor microenvironment promote therapy resistance through their pro-survival and immunosuppressive properties (55). Furthermore, the SASP factors secreted by senescent cells impact the tumor microenvironment, thereby influencing the efficacy of chemotherapy, radiation, and immunotherapy (56).

In conclusion, the relationship between senescence and tumor progression is intricate. Although senescence initially acts as a barrier against tumor development through OIS, the presence of SASP and altered immune surveillance associated with senescent cells can paradoxically promote tumor growth, invasion, and therapy resistance. A comprehensive understanding of the interplay between senescence and tumor progression is imperative for the development of targeted therapies that harness the tumor-suppressive aspects of senescence while mitigating its pro-tumorigenic effects.

## 4 Potential role of metabolic and senescence characteristics in ovarian cancer development and treatment

### 4.1 Potential role of metabolic characteristics

#### 4.1.1 Regulation of immune cell metabolism and function

Metabolic reprogramming plays a crucial role in the development and treatment of ovarian cancer. This reprogramming not only affects tumor cells but also exerts a significant influence on immune cell metabolism and function within the tumor microenvironment. An in-depth comprehension of the regulation of immune cell metabolism is imperative for the advancement of innovative therapeutic strategies that exploit the metabolic vulnerabilities specific to ovarian cancer.

Immune cells undergo metabolic adaptations to fulfill their distinct functions in the immune microenvironment. Notably, activated T cells exhibit a metabolic shift towards aerobic glycolysis, resembling that of tumor cells, in order to support their proliferation and effector functions. Glucose metabolism provides the necessary energy and biosynthetic intermediates for T cell activation, cytokine production, and cytotoxicity (23, 57). Metabolic regulation also affects the differentiation and function of other immune cell populations, such as DCs. DCs, which are pivotal in initiating immune responses, undergo metabolic changes to facilitate their antigen presentation capacity. Specifically, glycolysis and fatty acid oxidation are relied upon by DCs to generate energy and biosynthetic precursors for antigen processing and presentation (58, 59).

Moreover, metabolic alterations within the tumor microenvironment can impact immune cell function and promote immune evasion. Competition for limited nutrient resources between tumor cells and immune cells leads to nutrient deprivation and metabolic stress within the immune microenvironment. Consequently, this nutrient competition impairs immune cell metabolism, compromises immune cell activation, and suppresses anti-tumor immune responses (60, 61).

In addition to nutrient competition, metabolic waste products produced by tumor cells, such as lactate and adenosine, possess immunosuppressive effects.

The metabolic characteristics of immune cells in the tumor microenvironment can be targeted for therapeutic interventions as well. Strategies that enhance oxidative metabolism in T cells or inhibit glycolysis in immunosuppressive cells have demonstrated promise in preclinical models (27, 62).

Understanding the intricate interplay between metabolic reprogramming and immune cell function in ovarian cancer holds paramount importance in the development of effective therapeutic approaches. Targeting metabolic pathways in immune cells, mitigating nutrient competition, and disrupting immunosuppressive metabolic signaling represent potential avenues for augmenting the efficacy of immunotherapy and overcoming immune evasion in ovarian cancer.

#### 4.1.2 Targeted therapy of tumor cell metabolism

Current academic research has emphasized the potential of directing efforts towards tumor cell metabolism as a viable and promising strategy in the advancement and management of ovarian cancer. Distinct metabolic modifications serve as distinguishing features of cancer, including ovarian cancer, and precise targeting of specific metabolic pathways holds significant therapeutic promise. A notable metabolic alteration observed in ovarian cancer is the heightened occurrence of glycolysis, commonly known as the Warburg effect. Ovarian cancer cells exhibit escalated glucose uptake and glycolytic flux to sustain their rapid proliferation and survival. Promising results have been demonstrated in preclinical studies through the targeting of key enzymes involved in glycolysis, such as hexokinase and lactate dehydrogenase, which impedes tumor growth and increases the susceptibility of cancer cells to other treatment modalities (63, 64).

Another crucial metabolic pathway in ovarian cancer is fatty acid metabolism, wherein cancer cells utilize fatty acids as an energy source and as building blocks for membrane synthesis. Inhibition of pivotal enzymes engaged in fatty acid synthesis, such as acetyl-CoA carboxylase and fatty acid synthase, has exhibited anti-tumor effects in models of ovarian cancer (65, 66).

Moreover, the targeting of amino acid metabolism has emerged as a prospective therapeutic strategy. Ovarian cancer cells exhibit heightened uptake and utilization of amino acids to sustain their growth and survival. Inhibiting enzymes involved in amino acid metabolism, such as glutaminase and asparagine synthetase, has demonstrated promise in preclinical models by restricting tumor growth and sensitizing cancer cells to chemotherapy (67).

Additionally, the modulation of the tumor microenvironment to influence nutrient availability and metabolic signaling has shown potential. Disruption of angiogenesis, the process of new blood vessel formation, can restrict nutrient supply to the tumor and impair cancer cell metabolism. Consequently, the targeting of angiogenic pathways, including VEGF signaling, has been explored as a therapeutic approach in the context of ovarian cancer (68).

It is worth noting that the heterogeneous nature of ovarian cancer and the adaptability of metabolic pathways within the tumor necessitate a combination approach when targeting tumor cell metabolism. Current investigations involve combinations of metabolic inhibitors with chemotherapy, targeted therapies, or immunotherapies in order to enhance treatment effectiveness and overcome drug resistance (69).

In conclusion, the targeting of tumor cell metabolism presents a promising avenue for the advancement and management of ovarian cancer. Modulating glycolysis, fatty acid metabolism, amino acid metabolism, and angiogenesis hold potential for impeding tumor growth and increasing the sensitivity of cancer cells to therapy. Integration of metabolic inhibitors with other treatment modalities through combination strategies may yield synergistic effects and ultimately improve patient outcomes.

### 4.2 Potential role of senescence characteristics

#### 4.2.1 Immune cell senescence and the effect of immunotherapy

Immune cell senescence, characterized by irreversible cell cycle arrest and functional decline, exerts a multifaceted influence on ovarian cancer progression and therapeutic approaches. While senescent immune cells have been implicated in tumor promotion, they can also contribute to anti-tumor immune responses. Comprehending the impact of immune cell senescence on ovarian cancer and the potential for targeting senescent cells in immunotherapy is crucial for the development of effective treatment strategies. Accumulation of senescent immune cells, including senescent T cells and NK cells, occurs within the ovarian cancer tumor microenvironment, accompanied by phenotypic and functional alterations. Senescent T cells display reduced proliferation, impaired cytotoxicity, and altered cytokine secretion, leading to compromised anti-tumor activity. Similarly, senescent NK cells exhibit diminished cytotoxicity and cytokine production, impairing their ability to eliminate cancer cells (70). The presence of senescent immune cells in ovarian cancer is associated with immunosuppression and tumor progression. These cells secrete various factors, including components of the SASP, which promote tumor growth, angiogenesis, and immune evasion. SASP factors like interleukin-6 (IL-6) and tTGF-β contribute to the immunosuppressive microenvironment by recruiting Tregs and MDSCs (71, 72).

Despite their tumor-promoting effects, senescent immune cells can also confer benefits in ovarian cancer. Senescent immune cells positive for senescence-associated β-galactosidase (SA-β-gal) can enhance the priming of tumor-specific T cell responses and facilitate the recruitment of effector T cells to the tumor site. Additionally, senescent immune cells secrete chemokines that attract immune effector cells, potentially promoting anti-tumor immune responses (73, 74). Immunotherapy has emerged as a promising avenue for treating ovarian cancer, and researchers are exploring the impact of immune cell senescence on immunotherapeutic strategies. Immune checkpoint inhibitors, such as antibodies targeting PD-1, have demonstrated efficacy in various cancers, including ovarian cancer. However, the presence of senescent immune cells and the immunosuppressive microenvironment can restrict the response to immunotherapy (75, 76). Combining immunotherapy with strategies targeting senescent immune cells holds promise for improving treatment outcomes (**Table 1**). Selective elimination or rejuvenation of senescent immune cells, using senolytic agents or senescence-reversing interventions, respectively, may enhance the effectiveness of immunotherapy by reducing immunosuppression and restoring immune cell function (77).

**Table 1:** The metabolic agents for the treatment of ovarian cancer.

Target	Metabolic agent	Effect	Satge and subjects
V-ATPases	bafilomycin A1	Inhibition proliferation and migration	Preclinical study in cells
Proton pump	esomeprazole (EMSO)	Increasing the sensitivity to PTX, reducing autophagy; promoting apoptosis.	Preclinical study in cells
HIF-1α	2-methoxyestradiol	Anti-angiogenesis and growth	Hoosier OncologyGroup trial in patients with recurrent, platinum-resistant EOC.
mTOR	Temsirolimus	Limiting the proliferation and progression of ovarian cancer, enhancement of bevacizumab viability	Phase I study in patients with gynecologic cancers (54% weith ovarian cancer)
MCTs	Lonidamine	Inhibition of the growth and DNA repair process of ovarian cancer cells and enhancement of cisplatin/carboplatin/paclitaxel viability	Phase II study in patients with advanced ovarian cancer
Glutaminase	CB-839	Induction of oxidative and replication stress, acting synergistically with PDL1	Preclinical study in cells and mouse model

HIF-1α, Hypoxia-inducible factor-1; mTOR, Mammalian target of rapamycin; MCTs, Monocarboxylate transporters.

In conclusion, immune cell senescence in the context of ovarian cancer elicits both tumor-promoting and anti-tumor effects. Understanding the intricate interplay among senescent immune cells, the tumor microenvironment, and immunotherapy is imperative for the development of effective treatment strategies. Targeting senescent immune cells and modulating the immunosuppressive microenvironment may enhance the outcomes of immunotherapeutic interventions in ovarian cancer.

#### 4.2.2 Association between tumor cell senescence and treatment resistance

Tumor cell senescence, a state of permanent growth arrest, has been implicated in the emergence of treatment resistance in ovarian cancer. A growing body of evidence suggests that senescent tumor cells play a role in therapy resistance through diverse mechanisms. Gaining a comprehensive understanding of the link between tumor cell senescence and treatment resistance is crucial for advancing therapeutic strategies in ovarian cancer. Senescent tumor cells exhibit altered characteristics and release a variety of factors collectively referred to as the SASP. The SASP includes pro-inflammatory cytokines, growth factors, and enzymes involved in remodeling the extracellular matrix. These factors can promote cancer cell survival, evasion of the immune system, and resistance to therapy. Consequently, they contribute to the development of a tumor-supportive microenvironment that facilitates tumor cell survival and treatment resistance (71).

One of the mechanisms through which senescent tumor cells foster treatment resistance is by inducing a senescence-associated quiescent state. Senescent cells can enter a dormant state characterized by reduced metabolic activity and heightened resistance to chemotherapy. This quiescent state enables tumor cells to evade the cytotoxic effects of chemotherapy drugs, leading to treatment resistance and the recurrence of the disease (78, 79). Senescent tumor cells can also activate survival signaling pathways, such as the PI3K/Akt and MAPK/ERK pathways, which are associated with resistance to chemotherapy and targeted therapies. These signaling pathways promote cell survival, DNA repair, and the efflux of drugs, thus contributing to treatment resistance and limiting the effectiveness of cancer therapies.

Furthermore, senescent tumor cells can undergo epithelial-mesenchymal transition (EMT), a process linked to increased invasiveness, metastasis, and resistance to chemotherapy. EMT empowers tumor cells to acquire stem-like properties, enhance drug efflux, and evade immune surveillance, thereby causing therapy resistance and disease progression (80, 81). Additionally, senescence-induced alterations in the tumor microenvironment can contribute to treatment resistance. Senescent tumor cells have the ability to modulate the immune microenvironment, promoting the recruitment of immunosuppressive cells. These immunosuppressive cells establish an immune milieu that suppresses anti-tumor immune responses and impedes the effectiveness of immunotherapies (82, 83). By understanding the association between tumor cell senescence and treatment resistance, we can gain insights into potential therapeutic strategies. Targeting senescent tumor cells or modulating the associated pathways could overcome treatment resistance and enhance patient outcomes. Strategies such as senolytic therapy, which selectively eliminates senescent cells, or combination therapies that target both senescent tumor cells and their microenvironment, hold promise for overcoming treatment resistance and improving the efficacy of ovarian cancer therapies (84, 85).

Tumor cell senescence is closely linked to treatment resistance in ovarian cancer. Senescent tumor cells contribute to therapy resistance through various mechanisms, including the induction of a quiescent state, activation of survival signaling pathways, promotion of EMT, and modulation of the tumor microenvironment. Understanding and targeting these senescence-associated mechanisms may present novel opportunities to overcome treatment resistance and enhance therapeutic outcomes in ovarian cancer.

### 4.3 Metabolic influence on senescence in immune cells within the ovarian cancer tumor microenvironment

The metabolic milieu of the tumor microenvironment in ovarian cancer exerts a significant influence on the induction of senescence in immune cells. The scarcity of nutrients and oxygen, coupled with the acidic and hypoxic conditions, can incite immune cells (86). Tumor cells often secrete metabolically demanding factors, further exacerbating nutrient competition. Dysfunctional mitochondria in immune cells due to these unfavorable conditions can also trigger senescence.

Senescent immune cells within the tumor microenvironment often exhibit a pro-inflammatory phenotype, secreting cytokines and chemokines. These senescence-associated factors can promote chronic inflammation, favoring tumor growth, angiogenesis, and immune evasion (87, 88). Moreover, senescent immune cells might lose their cytotoxic functions, impairing their ability to target tumor cells effectively. Senescent immune cells may lose their ability to proliferate and become less responsive to antigens. This, in turn, compromises the antitumor immune response. Additionally, the secretion of pro-inflammatory factors by senescent immune cells can contribute to the immunosuppressive character of the tumor microenvironment (89, 90).

Understanding the intricate interplay between the metabolic state, senescence induction in immune cells, and their subsequent effects on both tumor and immune cells is pivotal for developing targeted therapeutic approaches in the context of ovarian cancer.

## 5 Research progress and future prospects of immunotherapy for ovarian cancer

### 5.1 Advances in immunotherapy of ovarian cancer

Immunotherapy has emerged as a promising therapeutic modality in the realm of ovarian cancer treatment, presenting novel opportunities to enhance patient outcomes. This section aims to elucidate recent advancements in immunotherapeutic strategies for ovarian cancer, encompassing immune checkpoint inhibitors, adoptive cell therapies, cancer vaccines, and combination approaches.

Targeting PD-1 and PD-L1, immune checkpoint inhibitors have exhibited clinical efficacy among specific subsets of ovarian cancer patients. Clinical trials have revealed enduring responses and augmented survival rates in patients receiving PD-1/PD-L1 blockade, both as a monotherapy and in conjunction with other agents (8, 91). Significant studies have explored the utilization of pembrolizumab, nivolumab, and atezolizumab within advanced ovarian cancer contexts. Adoptive cell therapies, including chimeric antigen receptor (CAR) T cell therapy and tumor-infiltrating lymphocyte (TIL) therapy, have demonstrated promise for ovarian cancer treatment. CAR T cell therapies that target tumor-associated antigens, such as mesothelin and folate receptor alpha, have shown encouraging outcomes in early-phase clinical trials (92). TIL therapy, involving the isolation and expansion of tumor-infiltrating lymphocytes derived from patient tumors, exhibits potential in generating tumor-specific immune responses (93). Cancer vaccines endeavor to activate the patient’s immune system against tumor antigens. In ovarian cancer, therapeutic vaccines targeting tumor-associated antigens, such as NY-ESO-1 and WT1, have been the subject of investigation in clinical trials. These vaccines are capable of eliciting antigen-specific immune responses and have displayed potential when employed in conjunction with other immunotherapies or chemotherapy (94, 95). The integration of diverse immunotherapy strategies or the amalgamation of immunotherapy with standard treatments has garnered attention in the context of ovarian cancer. Combinations of immune checkpoint inhibitors with chemotherapy, targeted therapies, or other immunotherapies strive to augment antitumor immune responses and surmount resistance mechanisms (96, 97). Ongoing preclinical and clinical investigations aim to identify optimal combination regimens. The identification of predictive biomarkers to discern patients likely to respond to immunotherapy assumes critical importance. PD-L1 expression on tumor cells and tumor-infiltrating immune cells has been explored as a prospective biomarker in ovarian cancer, although its predictive value remains a subject of controversy (98, 99). Other immune-related biomarkers, such as tumor mutational burden and immune cell infiltrates, are under exploration to refine patient selection criteria and enhance treatment outcomes.

In conclusion, immunotherapy exhibits promise as a modality for ovarian cancer treatment. Immune checkpoint inhibitors, adoptive cell therapies, cancer vaccines, and combination approaches present novel avenues to enhance patient outcomes. Further research endeavors should focus on optimizing immunotherapy strategies, identifying predictive biomarkers, and surmounting resistance mechanisms, with the ultimate goal of providing more efficacious and personalized treatments for patients with ovarian cancer.

### 5.2 Future research directions and challenges for immunotherapy of ovarian cancer

Despite the encouraging outcomes observed in ovarian cancer immunotherapy, there exist several challenges and areas warranting further investigation. In accordance with extant academic research, this section aims to delineate prospective research avenues and challenges concerning immunotherapy in the context of ovarian cancer.

The persistence of immunotherapy resistance represents a significant hurdle in ovarian cancer treatment. Tumor cells employ diverse mechanisms to elude immune surveillance, encompassing the upregulation of immune checkpoint molecules, immunosuppressive cell populations, and impairment of immune cell functionality. To augment the efficacy of immunotherapy, comprehending these resistance mechanisms and formulating strategies to surmount them is imperative (100). A primary priority lies in the identification of dependable biomarkers that can predict treatment response and facilitate the selection of patients who are most likely to benefit from immunotherapy. Although current biomarkers such as PD-L1 expression have displayed inconsistent predictive value in ovarian cancer, future research should concentrate on identifying novel biomarkers, including immune-related genetic signatures, tumor mutational burden, and immune cell profiles. These biomarkers can guide patient selection and aid in treatment decision-making (101, 102). The integration of immunotherapies with other treatment modalities, such as chemotherapy, targeted therapies, and radiation, holds promise for fortifying antitumor immune responses and circumventing resistance. Optimal combination regimens, treatment sequencing, and timing necessitate investigation through preclinical and clinical studies to maximize therapeutic outcomes (103). An emerging research realm involves the development of personalized immunotherapy approaches tailored to individual patient characteristics. This encompasses leveraging patient-specific tumor antigens, adoptive cell therapies, and neoantigen-based vaccines. Capitalizing on patient-specific immunotherapies has the potential to elicit improved treatment responses and long-term remissions (104, 105). The intricate tumor microenvironment plays a pivotal role in modulating immune responses in ovarian cancer. Deciphering the cellular and molecular constituents of the tumor microenvironment, including immune cell subsets, stromal cells, and extracellular matrix components, is essential for unraveling intricate interactions and devising strategies to manipulate the microenvironment in favor of antitumor immunity (106, 107). Targeted therapies that selectively inhibit key oncogenic pathways have demonstrated efficacy in subsets of ovarian cancer patients. By combining these targeted therapies with immunotherapy, treatment responses can potentially be enhanced by synergistically targeting both tumor cell-intrinsic and immune cell-extrinsic factors (108, 109). As immunotherapy gains recognition as a standard ovarian cancer treatment option, ensuring the long-term safety and monitoring of patients becomes paramount. Research efforts should be directed towards comprehending and managing immune-related adverse events, evaluating the long-term effects on immune function, and developing strategies for achieving durable responses without excessive toxicity (110).

In conclusion, although immunotherapy has exhibited promising results in the treatment of ovarian cancer, further research is necessary to surmount resistance mechanisms, identify reliable biomarkers, optimize combination strategies, and devise personalized approaches. Additionally, comprehending the tumor microenvironment and exploring the integration of immunotherapy with targeted therapies constitute crucial research directions. Moreover, guaranteeing the long-term safety and monitoring of patients undergoing immunotherapy remains a challenge that warrants ongoing investigation.

## 6 Conclusion and discussion

Ovarian cancer is a complex disease with limited treatment options, and there is a growing interest in harnessing the immune system to improve patient outcomes. In this comprehensive review, we have discussed the metabolic and senescence characteristics associated with the immune microenvironment in ovarian cancer, as well as their implications for tumor development and treatment. We have explored the role of the immune microenvironment, the regulation of immune cell metabolism, the effects of tumor cell metabolism, the reprogramming of metabolic pathways, immune cell senescence, and the relationship between senescence and tumor progression. Additionally, we have examined the potential of these characteristics in ovarian cancer development and treatment, including their impact on immunotherapy and targeted therapies. Lastly, we have outlined the advances in immunotherapy, future research directions, and the challenges that lie ahead.

One of the key findings highlighted in this review is the critical role of the immune microenvironment in ovarian cancer. The tumor microenvironment is composed of various immune cell populations, stromal cells, and extracellular matrix components, which interact with tumor cells to shape the immune response. Understanding the composition and dynamics of the immune microenvironment is essential for designing effective immunotherapeutic strategies. It has been demonstrated that the immune microenvironment in ovarian cancer is characterized by an immunosuppressive phenotype, marked by the presence of regulatory T cells, myeloid-derived suppressor cells, and M2 macrophages. These immune cells create an inhibitory environment that hampers antitumor immune responses.

Furthermore, the metabolic reprogramming of immune cells and tumor cells within the immune microenvironment plays a crucial role in ovarian cancer progression. Immune cells, such as T cells and dendritic cells, rely on specific metabolic pathways to support their activation, proliferation, and effector functions. Tumor cells, on the other hand, exhibit altered metabolic profiles that promote their survival, growth, and immune evasion. By targeting these metabolic pathways, it is possible to modulate immune cell function and enhance antitumor immune responses.

Senescence, a state of irreversible cell cycle arrest, is another important characteristic of the immune microenvironment in ovarian cancer. Senescent immune cells exhibit altered functional properties, including decreased proliferation, impaired cytokine production, and altered immune surveillance. These senescent cells accumulate in the tumor microenvironment and contribute to immune dysfunction and tumor progression. Understanding the effects of senescence on immune cell function is crucial for developing strategies to overcome immune senescence and restore immune competence in ovarian cancer.

The implications of these characteristics for ovarian cancer treatment are significant. Immunotherapy has emerged as a promising therapeutic approach, and several clinical trials have demonstrated encouraging results. However, resistance mechanisms and limited patient selection criteria remain major challenges. Overcoming resistance mechanisms, identifying reliable biomarkers, and optimizing combination strategies are key areas of future research. Personalized immunotherapy approaches tailored to individual patients’ characteristics, such as patient-specific tumor antigens and neoantigen-based vaccines, hold promise for improving treatment outcomes.

Combining immunotherapy with targeted therapies is another avenue to explore. Targeted therapies that inhibit specific oncogenic pathways have shown efficacy in subsets of ovarian cancer patients. Combining these targeted therapies with immunotherapy has the potential to enhance treatment responses by synergistically targeting both tumor cell-intrinsic and immune cell-extrinsic factors.

Despite the advances in immunotherapy, there are still challenges to address. Long-term safety and monitoring of patients receiving immunotherapy are essential, as immune-related adverse events can occur. Additionally, understanding the complex interplay between the tumor microenvironment and the immune system is crucial for designing effective therapeutic strategies.

In conclusion, the metabolic and senescence characteristics associated with the immune microenvironment in ovarian cancer have significant implications for tumor development and treatment. Understanding these characteristics and their interactions is essential for improving patient outcomes. Advances in immunotherapy, personalized approaches, and combination strategies hold promise for enhancing antitumor immune responses in ovarian cancer. However, further research is needed to overcome resistance mechanisms, identify reliable biomarkers, optimize treatment regimens, and ensure long-term safety and monitoring. By addressing these challenges, we can pave the way for more effective and personalized immunotherapeutic approaches for ovarian cancer patients.

## Author contributions

JX: Funding acquisition, Writing – original draft. YF: Writing – original draft. JH: Writing – review & editing. YW: Writing – review & editing. XJ: Software, Visualization, Writing – review & editing. XW: Funding acquisition, Visualization, Writing – review & editing. LH: Conceptualization, Writing – review & editing. ZH: Conceptualization, Project administration, Writing – review & editing.
